# Comprehensive Analysis of Prognostic Value and Immune Infiltration of IGFBP Family Members in Glioblastoma

**DOI:** 10.1155/2022/2929695

**Published:** 2022-07-04

**Authors:** Zhenglan Zhong, Xiaoping Xu, Shiguo Han, Yongxiang Shao, Yong Yi

**Affiliations:** ^1^Department of Health Examination, The Second People's Hospital of Yibin, Yibin, Sichuan Province 644000, China; ^2^Department of Neurosurgery, The Second People's Hospital of Yibin, Yibin, Sichuan Province 644000, China; ^3^Department of Neurosurgery, The Fifth People's Hospital of Yibin, Yibin, Sichuan Province 644000, China

## Abstract

Glioblastoma (GBM) is the most common primary malignant brain tumor in adults. The insulin-like growth factor-binding protein (IGFBP) family is involved in tumorigenesis and the development of multiple cancers. However, little is known about the prognostic value and regulatory mechanisms of IGFBPs in GBM. Oncomine, Gene Expression Profiling Interactive Analysis, PrognoScan, cBioPortal, LinkedOmics, TIMER, and TISIDB were used to analyze the differential expression, prognostic value, genetic alteration, biological function, and immune cell infiltration of IGFBPs in GBM. We observed that IGFBP1, IGFBP2, IGFBP3, IGFBP4, and IGFBP5 mRNA expression was significantly upregulated in patients with GBM, whereas IGFBP6 was downregulated; this difference in mRNA expression was statistically insignificant. Subsequent investigations showed that IGFBP4 and IGFBP6 mRNA levels were significantly associated with overall survival in patients with GBM. Functional Gene Ontology Annotation and Kyoto Encyclopedia of Genes and Genomes pathway enrichment analysis revealed that genes coexpressed with IGFBP4 and IGFBP6 were mainly enriched in immune-related pathways. These results were validated using the TIMER and TSMIDB databases. This study demonstrated that the IGFBP family has prognostic value in patients with GBM. IGFBP4 and IGFBP6 are two members of the IGFBP family that had the highest prognostic value; thus, they have the potential to serve as survival predictors and immunotherapeutic targets in GBM.

## 1. Introduction

Glioblastoma (GBM) is the most common primary malignant tumor of the central nervous system in adults. According to the CBTRUS Statistical Report, the most frequently occurring central nervous system tumor reported in the United States between 2013 and 2017 was GBM, which constituted 14.5% and 48.6% of all tumors and malignant tumors, respectively [1]. Unfortunately, no treatment has significantly altered the clinical outcomes of GBM despite extensive efforts in basic, translational, and clinical research conducted over the past few decades [2]. In addition, the lack of specific and sensitive biomarkers represents a major barrier in the management of this tumor [3–5]. Furthermore, intratumoral heterogeneity [6]and the complex tumor microenvironment conditions contribute to the poor prognosis of GBM. An area of active research is the use of immunotherapy to promote antitumor immune responses; it is an attractive approach to treating GBM [7]. The development of mechanism-based approaches and the identification of new therapeutic targets are therefore urgently needed.

The insulin-like growth factor binding protein (IGFBP) family comprises six identified members (IGFBP-1–6) [8], which are implicated in the pathophysiology process of numerous human diseases, including cancer [9]. Although the precise mechanism is not completely understood, the IGFBP family plays important roles in lung cancer [10], ovarian cancer [11], breast cancer [12], and obesity [13]. Using the polymerase chain reaction, GBM cell lines were found to express mRNA for IGFBP genes: IGFBP-1 in 42%, IGFBP-2 in 65%, IGFBP-3 in 97%, IGFBP-4 in 3%, IGFBP-5 in 74%, IGFBP-6 in 94%, and IGFBP-7 in 87% of glioma cell lines [14]. In addition, a number of previous studies have demonstrated that IGFBPs play an important role in tumorigenesis and the progression of glioma [15–20]. A study has also assessed the diagnostic roles of circulating IGFBP concentrations in GBM patients [21]. Nonetheless, the distinct roles of IGFBP family members in GBM, particularly in relation to tumor microenvironment and immune status, remain unclear. In this study, IGFBPs in GBM were comprehensively analyzed via various public databases to explore the expression, mutation, prognostic value, and immune cell infiltration.

## 2. Materials and Methods

### 2.1. Oncomine Database Analysis

The mRNA expression levels of IGFBP family members, implicated in various types of cancer, were evaluated in samples from cancer patients and healthy individuals (normal control) using the Oncomine database (https://www.oncomine.org/), which is a publicly accessible online platform that provides genome-wide expression analysis [22]. In this study, the thresholds were set as follows: *P* value <0.01, fold change = 2, and gene rank: top 10%.

### 2.2. Gene Expression Profiling Interactive Analysis (GEPIA)

The GEPIA database (https://gepia.cancer-pku.cn/index.html) is a web server that uses a standard processing pipeline for analysis and consists of RNA sequencing expression data of 9,736 tumors and 8,587 normal samples from The Cancer Genome Atlas (TCGA) and GTEx projects [23]. In this study, differential gene expression and correlative prognostic analyses were performed using the GEPIA database. The Student's *t*-test was used to generate *P* values for expression analysis. For further verification, survival analysis was performed using the Kaplan–Meier curve.

### 2.3. PrognoScan Database Analysis

The PrognoScan database (https://kmplot.com/analysis/) is a tool used to investigate the prognostic value of genes [24]. We analyzed the correlation between the expression of IGFBPs and clinical prognosis in various types of cancer using the PrognoScan database. The analysis included all survival values, such as overall survival (OS), relapse-free survival, disease-free survival (DFS), distant metastasis-free survival, and disease-specific survival. Further analyses only included studies with corrected *P* < 0.05.

### 2.4. cBioPortal Database Analysis

The cBioPortal database (https://www.cbioportal.org) is a comprehensive web resource that provides visual and multidimensional cancer genomic data [25]. In this study, we explored the genetic alterations of IGFBPs in GBM samples from TCGA. Datasets from Mayo Clinic 2019, PanCancer Atlas, Firehose Legacy, Cell 2013, Columbia, Nat Med 2019, and Nature 2008 were selected for further analysis. In addition, we analyzed the correlation between IGFBP alterations and survival outcomes in patients with GBM using Kaplan–Meier plots. Differences in survival curves were analyzed using the log-rank test, and a *P* value  < 0.05 was considered statistically significant.

### 2.5. LinkedOmics Database Analysis

LinkedOmics is a public database (https://www.linkedomics.org) that contains multiomics data from all 32 TCGA cancer types and 10 Clinical Proteomics Tumor Analysis Consortium cancer cohorts [26]. We used the LinkFinder module to explore genes that were differentially expressed in correlation with IGFBP4 and IGFBP6, based on the TCGA GBM cohort (*N* = 153). The results were presented as heat maps. The Gene Set Enrichment Analysis tool in the LinkInterpreter module was used to perform Kyoto Encyclopedia of Genes and Genomes (KEGG) and Gene Ontology (GO) pathway analysis of the differentially expressed genes.

### 2.6. Timer Database Analysis

The Tumor Immune Estimation Resource database (TIMER, https://cistrome.shinyapps.io/timer/) is designed to systematically evaluate the infiltration of different immune cells and their clinical impact [27]. The “correlation module” was used to visualize the correlation between IGFBPs expressions and IGF1 in GBM. To visualize the correlation between IGFBP gene expression and immune infiltration level in GBM, the “gene module” was used to generate scatterplots.

### 2.7. TISIDB Database Analysis

We used the TISIDB database (https://cis.hku.hk/TISIDB) to further explore the role of IGFBPs in the relationship between GBM and the immune system. The TISIDB database is an online platform that uses literature mining and high-throughput data analysis to analyze interactions between the immune system and tumors; it integrates multiple heterogeneous data types and various resources of immunological data retrieved from seven public databases [28]. In the present study, the TISIDB database was used to analyze the correlations between IGFBP expression and 28 types of tumor-infiltrating lymphocytes (TILs), immunomodulators (immuno-inhibitors), and chemokines involved in human cancers. Spearman's correlation coefficients were used, and *P* values <0.05 were considered statistically significant.

## 3. Results

### 3.1. Differential Expression of IGFBPs

**Figure 1:**
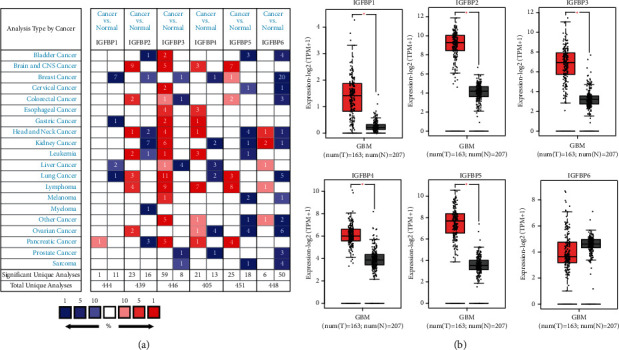
(a) IGFBP expression levels in different types of human cancers compared with normal tissues in the Oncomine database. (b) Validation of IGFBP expression between GBM samples and normal tissue from GEPIA.

**Figure 2:**
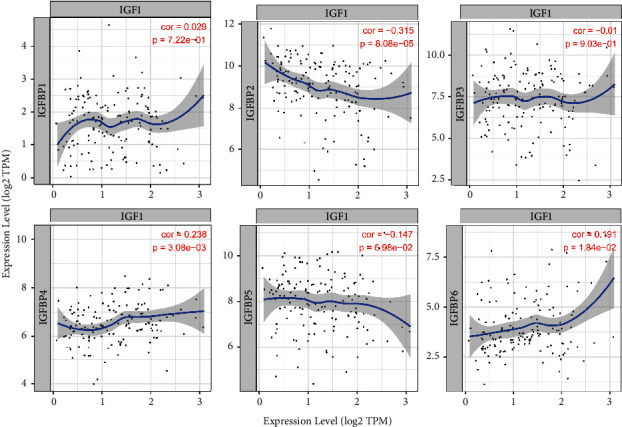
Correlation of the IGFBP expression level with IGF1 in GBM.

We first analyzed the transcriptional levels of IGFBPs in GBM and normal tissues using the Oncomine database. The results are shown in Figure 1(a). Based on the data from the Oncomine database, the transcriptional levels of IGFBP2, IGFBP3, IGFBP4, and IGFBP5 were observed to be elevated in brain and CNS cancer (Table 1). We further validated the expression of IGFBPs using theGEPIA database. We found that, with the exception of IGFBP6, the expression levels of the other five IGFBPs were significantly higher in GBM tissues than in normal tissues. Notably, the expression of IGFBP6 in GBM tissues was lower than that in normal tissues, but this difference was not statistically significant (Figure 1(b)). Thus, the results from the GEPIA database were consistent with the results from Oncomine database. As IGFBPs are key regulators of IGF-1, which is actively involved in tumorigenesis, we also analyzed the correlation between IGFBP family expressions and IGF-1 by using the TIMER database. Scatterplots showed a significant negative correlation between IGFBP2 expression and IGF-1, while a significant positive correlation was observed between IGFBP4 and IGFBP6 expressions and IGF-1 (Figure 2).

### 3.2. Prognostic Value of the mRNA Expression of IGFBPs

**Figure 3:**
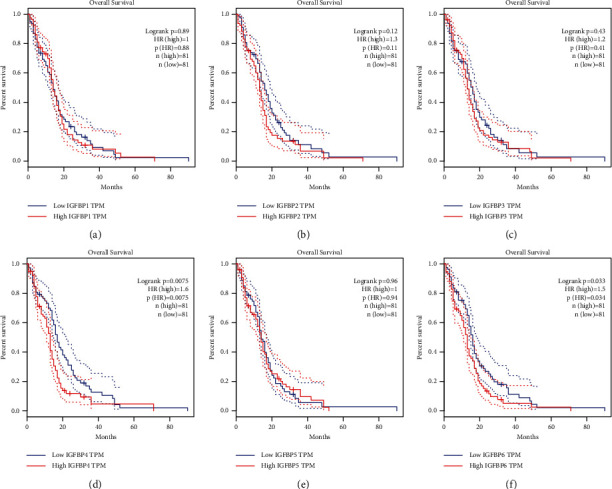
Prognostic value (OS) of the IGFBP expression in GBM (GEPIA).

**Figure 4:**
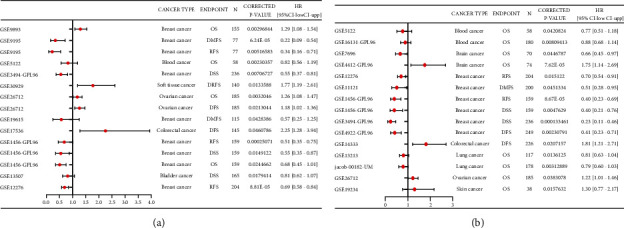
Forest plot displaying the prognostic results for IGFBP4 (a) and IGFBP6 (b) generated by using the PrognoScan database.

To evaluate the prognostic value of differentially expressed IGFBPs in patients with GBM, we assessed the correlation between differentially expressed IGFBPs and clinical outcomes using GEPIA. We found that GBM patients with low transcriptional levels of IGFBP4 (*P*=0.0075) and IGFBP6 (*P*=0.033) were significantly associated with longer overall survival (Figure 3), and we selected these for subsequent analysis. Thereafter, we used the PrognoScan database to investigate the prognostic value of IGFBP4 and IGFBP6 expression in patients with different types of cancer. The results are summarized in Figure 4. Notably, IGFBP6 expression was significantly correlated with the prognosis of seven types of cancer, including blood, breast, brain, colorectal, ovarian, lung, and skin cancer. We found that increased IGFBP6 expression was often associated with better prognosis in these types of cancer.

### 3.3. Genetic Alteration Analyses of IGFBPs

**Figure 5:**
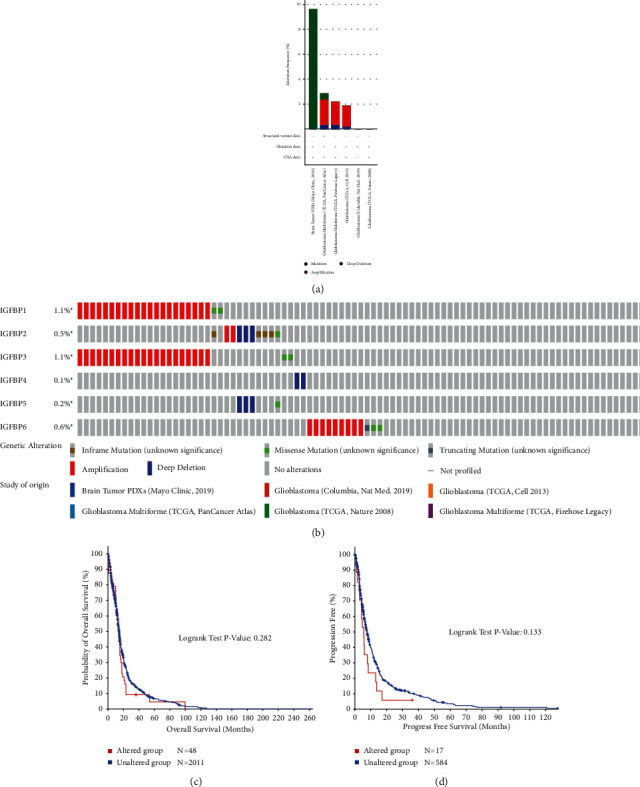
(a) Summary of IGFBPs alteration frequency in GBM. (b) OncoPrint visual summary of IGFBPs alterations. (c, d) Kaplan–Meier plots comparing OS (c) and PFS (d) in GBM patients with and without IGFBPs gene alterations.

We analyzed the genetic alterations in IGFBP genes using DNA sequencing data from GBM patients that was obtained from the cBioPortal online database. Six GBM datasets were analyzed, and the results showed the frequency of gene alterations, including mutations (0.51%–9.64%), amplifications (1.69%–2.03%), and deep deletions (0.19%–0.34%); mutations were the most frequently observed type of alteration (Figure 5(a)). The percentage of genetic alterations in specific IGFBP genes involved in GBM, ranged from 0.1% to 1.1% (Figure 5(b)). The prognostic value of IGFBPs, with or without genetic alterations, in GBM was analyzed, and no significant prognostic value was observed for OS (*P*=0.282) or DFS (*P*=0.133) (Figures 5(c) and 5(d)).

### 3.4. Functional Annotations and Predicted Signaling Pathways

**Figure 6:**
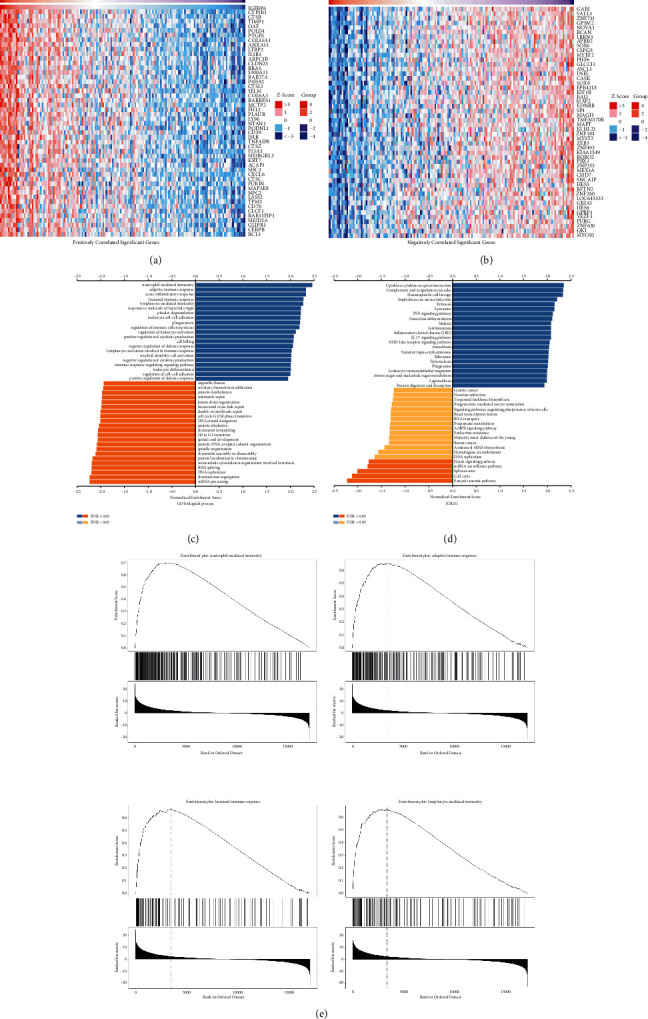
(a, b) Heatmaps of the top 50 genes positively and negatively correlated with IGFBP6 in TCGA-GBM. (c, d) GO annotations and KEGG pathways of IGFBP6 correlated genes in TCGA-GBM by GSEA. (e) Top four GO annotations of IGFBP6.

To further reveal the potential biological functions of IGFBPs in GBM, the LinkedOmics database, which contains data from TCGA, was used to analyze the mRNA sequencing data of patients with GBM. The heat map showed the top 50 significant genes that positively and negatively correlated with IGFBP6 and IGFBP4 expression. IGFBP6 expression showed a strong positive correlation with CYP1B1 (Pearson correlation = 0.715, *P*=2.8*e* − 25), CTSB (Pearson correlation = 0.700, *P*=7.15*e* − 24), and TIMP1 expression (Pearson correlation = 0.697, *P*=1.39*e* − 23) (Figure 6(a)). Additionally, there was a strong negative correlation between the expression of GAB1 (Pearson correlation = −0.695, *P*=2.21*e* − 23), SALL3 (Pearson correlation = −0.654, *P*=5.14*e* − 20), and ZNF711 (Pearson correlation = −0.646, *P*=2.01*e* − 19) (Figure 6(b)). The results of the top 50significant genes that were negatively and positively correlated with IGFBP4 are shown in Supplementary Figures 1A and B.

GO biological process analysis revealed significant correlation between the coexpressed genes and IGFBP6. Gene set enrichment analysis indicated that genes coexpressed with IGFBP6 were mainly involved in neutrophil-mediated immunity, adaptive immune response, acute inflammatory response, humoral immune response, and lymphocyte-mediated immunity(Figures 6(c) and 6(e)), while genes coexpressed with IGFBP4 were mainly involved in collagen metabolic processes, granulocyte activation, neutrophil-mediated immunity, adaptive immune response, and humoral immune response (Supplementary Figures 1C and E).

KEGG pathway analysis of the genes coexpressed with IGFBP6 showed enrichment in cytokine-cytokine receptor interaction, complement and coagulation cascades, Fanconi anemia pathway, and the cell cycle (Figure 6(d)); in contrast, genes coexpressed with IGFBP4 were enriched in collagen metabolic process, granulocyte activation, glutamate receptor signaling pathway, and brain morphogenesis (Supplementary Figure 1D). These results implied that IGFBP6 and IGFBP4 are involved in the modulation of various immune molecules in GBM and have an effect on immune cell infiltration of the tumor microenvironment.

### 3.5. The Association of IGFBP4 and IGFBP6 with Immune Cell Infiltration

**Figure 7:**
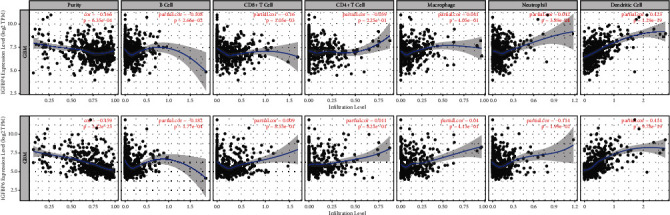
Correlation of IGFBP4 and IGFBP6 expression with immune infiltration level in GBM.

The TIMER database was used to evaluate whether the expression of IGFBP4 and IGFBP6 in GBM was correlated with immune cell infiltration. Interestingly, we found that, in GBM, the IGFBP4 expression level had a significant positive correlation with the level of infiltrating dendritic cells (*r* = 0.423, *P*=1.29*e* − 19) and a significant negative correlation with the level of tumor purity (*r* = –0.166, *P*=6.35*e* − 04), B cells (*r* = –0.108, *P*=2.66*e* − 02), and CD8^+^ T cells (*r* = –0.16, *P*=1.05*e* − 03). The IGFBP6 expression level had a significant positive correlation with the level of infiltrating dendritic cells (*r* = 0.414, *P*=9.78*e* − 19) and negatively correlation with the level of tumor purity (*r* = –0.459, *P*=3.22*e* − 23), B cells (*r* = –0.182, *P*=1.77*e* − 04), and neutrophils (*r* = –0.114, *P*=1.99*e* − 02) (Figure 7).

**Figure 8:**
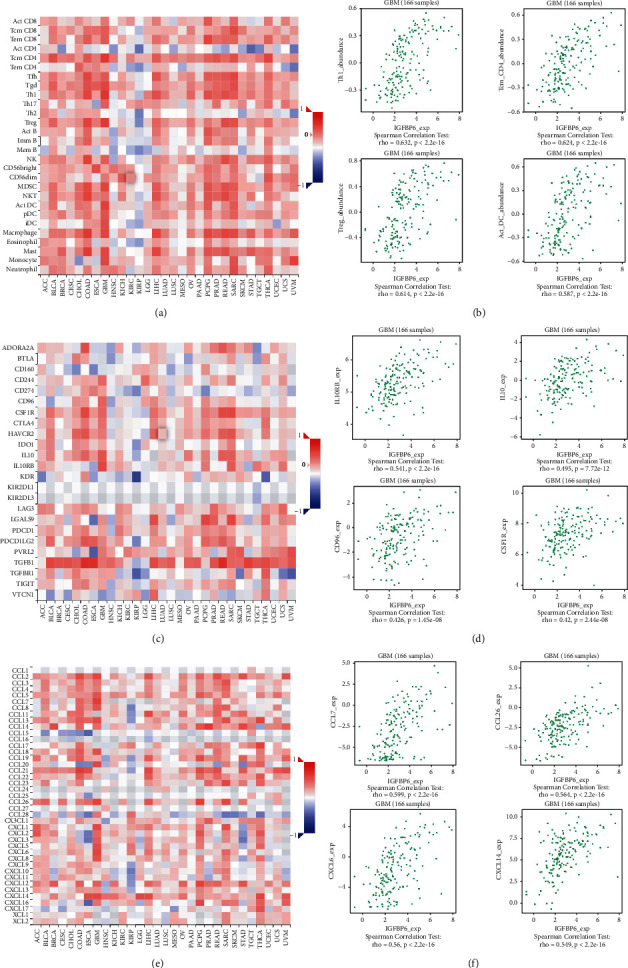
Associations of the IGFBP6 expression level with lymphocytes, immunomodulators, and chemokines in GBM from TISIDB database. (a) Correlations between abundance of tumour-infiltrating lymphocytes (TILs) and IGFBP6. (b) Four TILs with the highest correlation. (c) Correlations between immunomodulators and IGFBP6. (d) Four immunomodulators with highest correlation. (e) Correlations between chemokines and IGFBP6. (f) Four chemokines with the highest correlation.

A Cox proportional hazard model was constructed to evaluate the clinical relevance of immune cells in GBM. Additionally, a univariate Cox survival analysis demonstrated that only dendritic cell infiltration was significantly associated with the OS of patients with GBM (Table 2). Furthermore, a multivariate Cox survival analysis revealed that age, IGFBP6 expression, and IGFBP2 expression were independent prognostic biomarkers for GBM (Table 3). These findings suggested that IGFBP6 plays an important immune-related role in GBM. Finally, to further study the correlation between IGFBPs and immune infiltration, we investigated the relationship between IGFBP6 and IGFBP4 expression and various immune signatures using the TISIDB database. The TISIDB database includes data on tumor-infiltrating lymphocytes, immunomodulators, and chemokines. Using the TISIDB database, we found that the IGFBP6 expression correlated with TILs in GBM. The relationship between IGFBP6 expression and TILs in different types of cancer is shown in Figure 8(a). In GBM, the four TILs that had the strongest correlation with IGFBP6 were type 1 T helper cells (Th1, Spearman: *r* = 0.632, *P* < 2.2*e* − 16), central memory CD4 T cells (Tcm_CD4; Spearman: *r* = 0.624, *P* < 2.2*e* − 16), regulatory T cells (Treg, Spearman: *r* = 0.614, *P* < 2.2*e* − 16), and activated dendritic cells (Act_DC, Spearman: *r* = 0.587, *P* < 2.2*e* − 16) (Figure 8(b)). The correlation between the expression level of IGFBP6 and immuno-inhibitors is shown in Figure 8(c). Among the immuno-inhibitors, IL10RB (Spearman: rho = 0.541, *P* < 2.2*e* − 16), IL10 (Spearman: rho = 0.495, *P* < 7.72*e* − 12), CD96 (Spearman: rho = 0.426, *P* < 1.45*e* − 08), and CSF1R (Spearman: rho = 0.42, *P* < 2.44*e* − 08) (Figure 8(d)) showed the strongest correlation with IGFBP6 expression. The correlation between IGFBP6 expression and chemokines is shown in Figures 8(e) and 8(f) shows the correlation of IGFBP6 with CCL7 (Spearman's rho = 0.599, *P* < 2.2*e* − 16), CCL26 (Spearman's rho = 0.5645, *P* < 2.2*e* − 16), CXCL6 (Spearman's rho = 0.56, *P* < 2.2*e* − 16), and CXCL14 (Spearman's rho = 0.549, *P* < 2.2*e* − 16). The correlation between IGFBP4 expression and various immune signatures is shown in Supplementary Figure 2.

These results further confirmed that IGFBP6 and IGFBP4 are correlated with immune infiltrating cells in GBM, which suggests that IGFBPs, particularly IGFBP6, play a vital role in immune escape in the GBM tumor microenvironment.

## 4. Discussion

GBM is a lethal primary brain tumor. The treatment outcomes for patients with GBM have not improved over the past few decades. The molecular mechanism underlying the role of the IGFBP family in the tumorigenesis of GBM is still unclear. In this study, IGFBPs implicated in GBM were comprehensively analyzed in terms of expression, prognostic value, mutation, biological function, and immune cell infiltration. The results from the Oncomine database showed that IGFBP2, IGFBP3, IGFBP4, and IGFBP5 were expressed at a higher level in tissues from patients with brain and CNS cancer than in normal tissues from healthy individuals. This observation is consistent with findings from the GEPIA database. Analysis of the prognostic value showed that differential expression of IGFBP4 and IGFBP6 was significantly correlated with overall survival of GBM patients. The occurrence of IGFBP alterations in GBM was low. Additionally, there were no significant differences between OS and DFS of GBM patients with or without IGFBPs alterations. GO annotation and KEGG pathway enrichment analyses implied that IGFBP4 and IGFBP6 had immune-related functions, and this was validated using the TIMER and TSMIDB databases. Overall, these results suggest that IGFBP4 and IGFBP6 serve as valuable prognostic biomarkers and potential immunotherapeutic targets in GBM.

Owing to the lack of T-cell infiltration and the profound immunosuppressive nature of GBM, GBM is characterized as a “cold tumor.” Immunological treatment of cold tumors is a great challenge because of the absence of the adaptive immune response in immune cell infiltration [29, 30]. Despite the poor outcomes of previously developed immunotherapy treatments, there has been new preclinical and clinical developments in immunotherapy that are providing promising treatments for GBM [7].

In this study, our initial screening revealed that IGFBP4 and IGFBP6, of the IGFBP family, were related to the clinical outcomes of GBM. IGFBP4 has been consistently shown to inhibit insulin-like growth factor activity [31]. It has a growth inhibitory role and has been reported as one of the genes that is downregulated in colon cancer [32], breast cancer [33], and lung carcinoma [34]. Furthermore, it was reported that the IGFBP4 expression has tumor promoting effects in renal cell carcinoma [35] and glioma [36], suggesting a possible oncogenic role for IGFBP4. Several studies have shown that IGFBP4 promotes cell proliferation in GBM, epithelial-to-mesenchymal transition, and invasive cell migration and invasion [37] and inhibits the angiogenic response [38]. Nur et al. [39] assessed 83 volunteers (60 patients with lung cancer and 23 healthy individuals) and found that the serum concentration of IGFBP4 was a better predictor of lung cancer than serum concentrations of PAPP-A. In our study, high IGFBP4 mRNA expression was significantly correlated with low OS in patients with GBM, and function analysis suggested that IGFBP4 is strongly associated with immune cells, which is consistent with findings from previous studies. However, little is known about the immune-related function of IGFBP4. Recently, a whole-genome expression profiling study on postmortem brain tissue has provided insight into the correlation between IGFBP4 and immune responses [40]. Further investigations are required to verify these findings.

IGFBP6 has an affinity for IGF-II that is 20–100 fold higher than its affinity for IGF-I, and it has a highly conserved structure across species [41]. It was believed that the major function of IGFBP6 was inhibiting IGF-II-induced cell proliferation, migration, and survival [42]. The relationship between IGFBP6 and cancer remains unclear [9]. Notably, IGFBP6 has antitumor effects in many types of cancer, including neuroblastoma [43], colon [44], ovarian [45], and prostate cancer [46]. Moreover, many studies have demonstrated the downregulation of IGFBP6 expression in cancer; in contrast, several studies have shown upregulation of IGFBP6 in pancreatic cancer [47] and adrenocortical cancer [48]. Recently, it has been reported that IGFBP6 may exert immunological functions [49, 50]. IGFBP6 was found to be highly expressed in eosinophils [51], and it has also been reported that IGFBP6 is associated with allergic asthma [52] and thymic atrophy [53]. In rheumatoid arthritis patients, IGFBP6 was shown to be able to induce high levels of T-lymphocyte migration in vitro [54]. These evidences suggest an important role for IGFBP6 in immunity. IGFBP6 had the highest proportion of mRNA (94%) expressed in glioma cell lines that were derived from primary glioblastomas [14], and the biological function study showed that IGFBP6 is an unfavorable prognostic factor of patients with glioma [55]. The findings of the present study are consistent with the previous studies, and the expression of IGFBP6 was lower in tissues from patients with GBM than in normal tissues from healthy individuals (normal controls), which suggests that IGFBP6 has a significant effect on the prognosis of GBM. Functional analysis of IGFBP6 coexpressed genes revealed that the genes coexpressed with IGFBP6 were related to the immune system, indicating that IGFBP6 could not only serve as a potential prognostic indicator but may also be a new immune therapeutic target in GBM.

Our current study provides evidence that the dysregulation of IGFBP4 and IGFBP6 plays a vital role in the immune regulation of GBM, and this suggests a potential molecular mechanism in the progression of GBM that involves the IGFBP family.

## 5. Conclusions

In conclusion, our study's findings indicate that the IGFBP family has a prognostic role in GBM. IGFBP4 and IGFBP6 may serve as valuable prognostic indictors and potential immunotherapeutic targets in GBM. However, there are certain limitations that must be considered when interpreting our study's findings. The data analyzed in this study were obtained from online databases. Furthermore, the mechanisms by which IGFBP4 and IGFBP6 are involved in tumorigenesis and progression of GBM, especially immune regulation, require in vitro and in vivo studies.

## Figures and Tables

**Table 1:** Significant upregulated expression in mRNA level of IGFBPs between glioblastoma and normal tissues by Oncomine database.

Gene	*P* value	Fold change	*T*-test	Reference
IGFBP2	6.70*E* − 9	7.009	8.522	Shai brain (42)
	3.27*E* − 23	31.755	15.163	Sun brain (180)
	6.36*E* − 12	6.962	11.068	Bredel brain 2 (54)
	7.45*E* − 11	13.538	20.347	TCGA brain (557)
	1.04*E* − 5	5.576	9.551	Murat brain (84)

IGFBP3	2.70*E* − 20	8.562	11.865	Sun brain (180)
	9.24*E* − 8	70.634	18.998	Lee brain (101)
	1.55*E* − 6	5.536	6.328	Bredel brain 2 (54)
	3.93*E* − 5	2.372	7.042	Murat brain (84)
	9.13*E* − 5	1.845	4.235	Shai brain (42)
	0.028	4.368	3.179	Liang brain (38)
	0.011	4.387	3.297	TCGA brain (557)

IGFBP4	3.04*E* − 7	2.947	6.519	Shai brain (42)
	5.19*E* − 9	2.886	6.812	Sun brain (180)
	0.017	2.458	2.801	TCGA brain (557)
	0.007	3.721	5.279	Lee brain (101)
	0.011	1.243	3.269	Murat brain (84)

IGFBP5	2.14*E* − 12	6.647	10.863	Liang brain (38)
	2.56–11	15.589	19.599	Lee brain (101)
	1.92*E* − 9	4.492	9.673	Bredel brain 2 (54)
	1.13*E* − 5	3.883	5.902	Shai brain (42)
	2.52*E* − 14	2.417	8.809	Sun brain (180)
	2.88*E* − 10	1.932	8.692	Murat brain (84)
	0.002	3.678	4.726	TCGA brain (557)

**Table 2:** Univariate analysis of the correlation of IGFBPs expression and immune infiltrates with OS inGBM.

Cancer	Variable	*P*
GBM	IGFBP3	0.001584
GBM	Dendritic cell	0.001617
GBM	IGFBP5	0.003962
GBM	IGFBP2	0.004553
GBM	IGFBP6	0.02552
GBM	B cell	0.105243
GBM	IGFBP4	0.398653
GBM	CD4+ T cell	0.629122
GBM	IGFBP1	0.657062
GBM	Neutrophil	0.741002
GBM	Macrophage	0.755369
GBM	CD8+ T cell	0.805153

**Table 3:** Multivariate analysis of the correlation of IGFBPs expression and immune infiltrates with OS in GBM.

	Coef	HR (95%CI_l−95%CI_u)	*P* value	Sig
Age	0.027	1.027(1.018–1.036)	0.000	^*∗∗∗*^
IGFBP6	0.146	1.158 (1.036–1.293)	0.010	^*∗*^
IGFBP2	0.110	1.117 (1.016–1.227)	0.022	^*∗*^
Dendritic	0.384	1.468 (0.964–2.238)	0.074	
raceWhite	0.603	1.828 (0.739–4.523)	0.192	
IGFBP4	−0.084	0.920 (0.809–1.045)	0.200	
B cell	−0.425	0.654 (0.339–1.261)	0.205	
Neutrophil	0.608	1.836 (0.675–4.992)	0.234	
Gender (male)	0.079	1.082(0.859–1.363)	0.504	
CD8_T cell	0.156	1.169 (0.704–1.943)	0.546	
IGFBP1	−0.040	0.961 (0.842–1.097)	0.554	
Purity	0.211	1.235 (0.610–2.503)	0.557	
IGFBP3	−0.025	0.975 (0.893–1.065)	0.572	
Macrophage	0.231	1.259 (0.565–2.809)	0.573	
raceBlack	0.234	1.264(0.454–3.522)	0.654	
CD4_T cell	−0.082	0.922 (0.398–2.133)	0.849	
IGFBP5	0.002	1.002 (0.869–1.154)	0.982	

^*∗*^ *P* < 0.01; ^*∗∗∗*^*P* < 0.0001.

## Data Availability

Our study is based on public databases. Users can download relevant data for free for research and publish relevant articles.

## Supplementary Materials

Supplementary Materials
*Supplementary Figure 1.* (A-B) Heatmaps of the top 50 genes positively and negatively correlated with IGFBP4 in TCGA-GBM. (C-D) GO annotations and KEGG pathways of IGFBP4 correlated genes in TCGA-GBM by GSEA. (E) Top four GO annotations of IGFBP4. *Supplementary Figure 2.* Associations of the IGFBP4 expression level with lymphocytes, immunomodulators, and chemokines in GBM from TISIDB database. (A) Correlations between abundance of tumour-infiltrating lymphocytes (TILs) and IGFBP4. (B) Four TILs with the highest correlation. (C) Correlations between immunomodulators and IGFBP4. (D) Four immunomodulators with the highest correlation. (E) Correlations between chemokines and IGFBP4. (F) Four chemokines with the highest correlation.
