# Supplementary figures and images for: Comprehensive Analysis of Prognostic Value and Immune Infiltration of IGFBP Family Members in Glioblastoma
Source: J Healthc Eng. 2022 Jul 4;2022:2929695. doi: 10.1155/2022/2929695 (PMC9273392; doi:10.1155/2022/2929695)

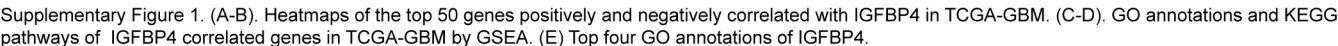

Supplement: Supplementary Materials — Supplementary Figure 1. (A-B) Heatmaps of the top 50 genes positively and negatively correlated with IGFBP4 in TCGA-GBM. (C-D) GO annotations and KEGG pathways of IGFBP4 correlated genes in TCGA-GBM by GSEA. (E) Top four GO annotations of IGFBP4. Supplementary Figure 2. Associations of the IGFBP4 expression level with lymphocytes, immunomodulators, and chemokines in GBM from TISIDB database. (A) Correlations between abundance of tumour-infiltrating lymphocytes (TILs) and IGFBP4. (B) Four TILs with the highest correlation. (C) Correlations between immunomodulators and IGFBP4. (D) Four immunomodulators with the highest correlation. (E) Correlations between chemokines and IGFBP4. (F) Four chemokines with the highest correlation. [file 2929695.f1.zip › 2929695.f1/Supplementary Figure 1.pdf]
